# Expression of the Human Herpesvirus 6A Latency-Associated Transcript U94A Disrupts Human Oligodendrocyte Progenitor Migration

**DOI:** 10.1038/s41598-017-04432-y

**Published:** 2017-06-21

**Authors:** Andrew Campbell, Jessica M. Hogestyn, Christopher J. Folts, Brittany Lopez, Christoph Pröschel, David Mock, Margot Mayer-Pröschel

**Affiliations:** 1 Department of Biomedical Genetics, University of Rochester, School of Medicine and Dentistry, 601 Elmwood Avenue, Box 633, NY, 14642 USA; 2 Environmental Health Science Center, University of Rochester, School of Medicine and Dentistry, 601 Elmwood Avenue, Box 633, Rochester, NY 14642 USA; 3 Department of Neuroscience, School of Medicine and Dentistry, University of Rochester, 601 Elmwood Avenue, Box 633, Rochester, NY 14642 USA; 4 Division of Newborn Medicine, Boston Children’s Hospital/Harvard Medical School, 300 Longwood Avenue, Boston, MA 02115 USA

## Abstract

Progression of demyelinating diseases is caused by an imbalance of two opposing processes: persistent destruction of myelin and myelin repair by differentiating oligodendrocyte progenitor cells (OPCs). Repair that cannot keep pace with destruction results in progressive loss of myelin. Viral infections have long been suspected to be involved in these processes but their specific role remains elusive. Here we describe a novel mechanism by which HHV-6A, a member of the human herpesvirus family, may contribute to inadequate myelin repair after injury.

## Introduction

A major challenge in treating demyelinating diseases is the frequent progression of patients from a relapsing-remitting state to a progressive chronic disease that is associated with a failure to repair myelin damage^1^. Effective myelin repair requires OPCs to undergo a highly controlled migration and differentiation response in a time and region-specific manner^2, 3^. While failures in the migration and differentiation of endogenous OPCs have been described, it is not clear why these functions are impaired in the context of demyelinating diseases and why some patients invariably progress to a chronic neurodegenerative stage^4, 5^.

Largely disregarded is the fact that the human CNS contains significant amounts of latent viruses, most of which are members of the herpesvirus family^6^. Infection with human herpesviruses 6 A (HHV-6A) in humans results in almost all cases in viral latency, a state where the virus does not produce a fully infectious virion^7^. HHV-6A latency can occur after a primary acute infection or through germline vertical transmission with the latter resulting in the presence of a copy of latent HHV-6A in every cell of the body^8^.

Establishment of latency of HHV-6A involves formation of a viral nuclear episome, integration into the human genome and expression of a limited number of viral transcript and proteins, the most prominent being U94A^9^. The latency state has generally been considered to be benign^10^, however, serological data from MS patients showing increased prevalence of anti-U94A immunoglobulins^11^ and the presence of viral genome, transcripts, and antigen in tissue samples from patients with demyelinating disease in the absence of infectious virions^12^ prompted us to consider the impact of latent HHV-6A on OPC functions that are relevant for repair processes. As primary infections mostly result in latency and production of U94A, we used expression of U94A in human OPCs (hOPCs) isolated from fetal human tissue as a model for latency.

## Results

**Figure 1:**
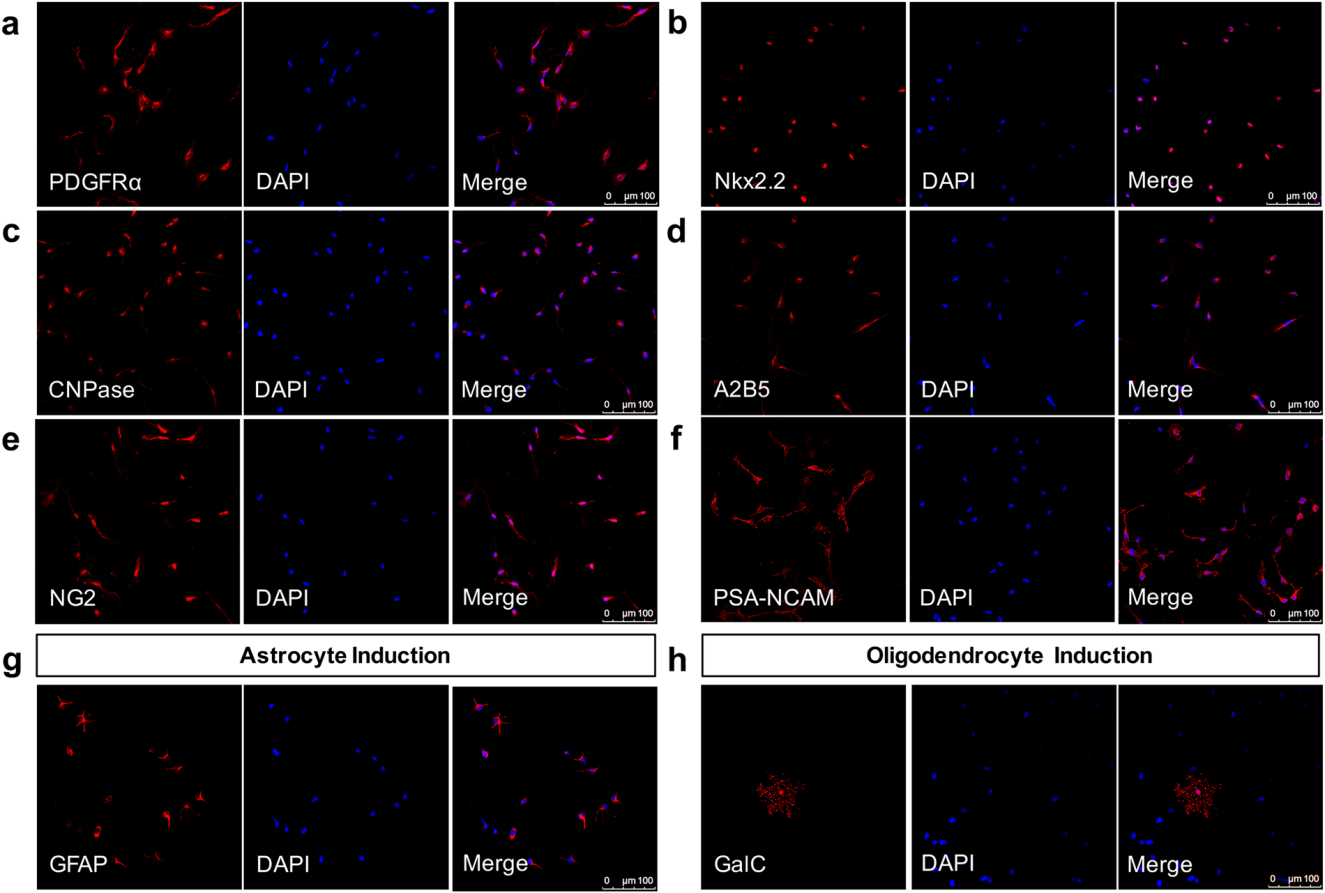
CD140+ hOPCs express common OPC markers and differentiate into oligodendrocytes and astrocytes *in vitro*. hOPCs were isolated from 19–22 week fetal human tissue by immunomagnetic purification using anti-CD140 beads. When cultured in the presence of PDGF and FGF for 7 days, these cells expressed a variety of known OPC markers, including (**a**) PDGFRα, (**b**) Nkx2.2, (**c**) CNPase, (**d**) A2B5, (**e**) NG2, and (**f**) PSA-NCAM. (**g**) When exposed to bone morphogenic protein 4 (BMP-4) for 7 days, cells differentiated into GFAP+ astrocytes. (**h**) When exposed to triiodothyronine/thyroxine (T_3_/T_4_) for 10 days, these cells differentiated into GalC+ oligodendrocytes.

**Figure 2:**
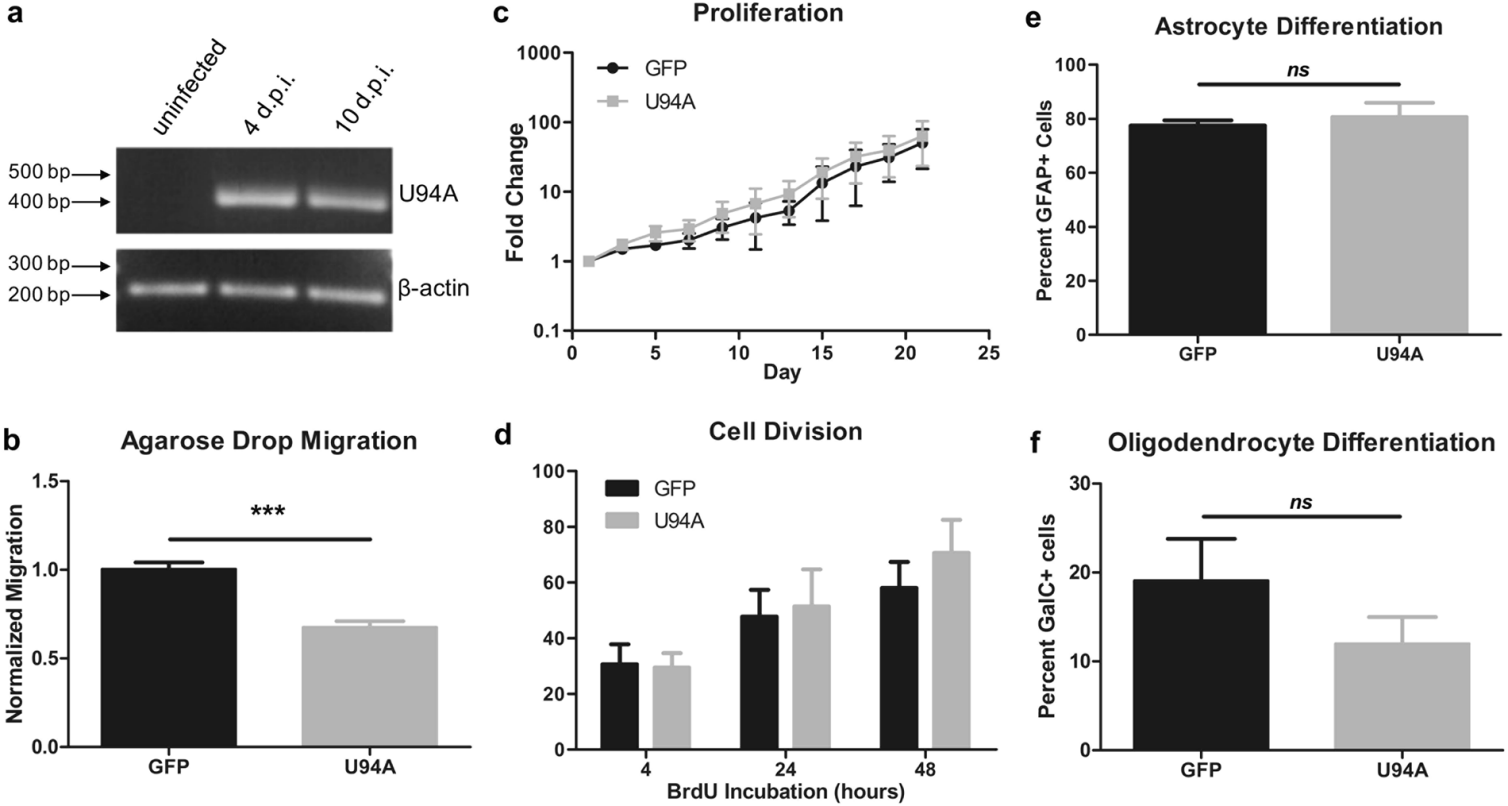
U94A expression inhibits hOPC migration *in vitro*. (**a**) Nested rtPCR shows that hOPCs infected with HHV-6A express U94A mRNA at 4 and 10 days post-infection. (**b**) Lentiviral expression of U94A RFP significantly inhibits hOPC migration as analyzed by an agarose drop assay. (**c**) U94A RFP expression does not affect hOPC proliferation over the course of 21 days *in vitro*. (**d**) Proportion of dividing hOPCs, determined by BrdU incorporation after 4, 24, and 48 hours of incubation. (**e**,**f**) U94A RFP expression does not significantly affect OPC differentiation into GFAP+ astrocytes or GalC+ oligodendrocytes *in vitro*. Data from 3–6 independent experiments, normalized to GFP+ controls, pooled and displayed as mean ± standard error of the mean (SEM); *ns = *not significant; ***p < 0.001 versus control. Effects of U94A expression on hOPC proliferation and BrdU incorporation were analyzed by two-way ANOVA. Effects of U94A expression on hOPC migration and differentiation were analyzed by unpaired student’s *t*-test. Nested rtPCR agarose gel shown as cropped image. Full images shown in supplementary information.

We previously showed that infection of hOPCs with whole virions of HHV-6A show syncytia formation, intercellular production of viral particles, cell cycle arrest and premature differentiation^13^. While it was at this time not known that HHV-6A establishes latency via genome integration, we noticed in these earlier studies that HHV-6A infected hOPCs did not die and the cell cycle arrest seemed transient, suggesting a possible progression from an abortive infection with no cytotoxic effects to a latency state. To test whether acutely infected hOPCs progress to such a latency stage and express the latency associated viral transcript U94A, we exposed hOPCs^14^ (Fig. 1) to cell-free, fluorescently labeled HHV-6A virions^13^ and, using reverse transcriptase PCR, detected robust expression of U94A at 4 and 10 days post-infection (Fig. 2a). As the cell cycle arrest that occurs after acute infection prevented us from generating large numbers of hOPCs that would express the latency transcript U94A, we directly expressed the latency gene in hOPCs using a lentiviral vector co-expressing red fluorescent protein (RFP). We then analyzed cell survival, migration, proliferation and differentiation, all of which are critical OPC functions during remyelination. hOPCs expressing only green fluorescent protein (GFP) served as controls. The expression of different fluorescent proteins in control versus U94A+ cells allowed us quantify the cellular readouts in experiments where we mixed the two populations to ensure exposure to identical conditions.

We found an intriguing impairment of *in vitro* migration in U94A-expressing hOPCs using an agarose drop assay. As quantified in (Fig. 2b) the number of U94A RFP+ hOPCs that migrated from the drop was significantly reduced compared to GFP+ control cells migrating from the same drop (p < 0.0001, unpaired t-test). We were concerned that the decrease in U94A+ cells that migrated out of the agarose drop could have been due to a decrease in cell survival of U94A+ cells. However, we did not see a significant change in the number of live cells of U94A+ versus control cultures (Fig. 2c) (p = 0.518, 2-way ANOVA). To confirm that the cell number data represent survival, rather than increased proliferation coupled with increased cell death, we labeled cells with BrdU and analyzed incorporation after 4, 24, and 48 hours (Fig. 2d). We did not find a significant difference in proportions of dividing cells, suggesting that the impaired migration of U94A+ hOPCs was not due to impaired survival or proliferation. To determine whether U94A expression affects differentiation, we induced cells with bone morphogenetic protein 4 (astrocyte induction) or thyroid hormone (oligodendrocyte induction). We found no significant differences in the number of astrocytes (Fig. 2e) or oligodendrocytes generated (Fig. 2f).

**Figure 3:**
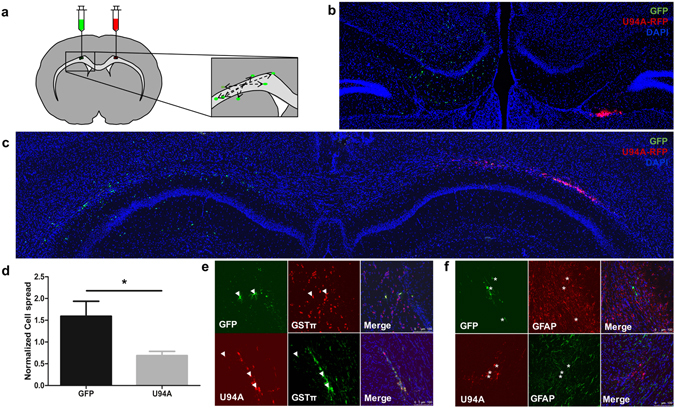
U94A expression inhibits hOPC migration *in vivo* but does not prevent oligodendrocyte differentiation. (**a**) hOPCs expressing U94A (RFP) or controls (GFP) were injected bilaterally into the corpus callosum and hippocampus of cuprizone-treated NSG mice. Brains were analyzed 21 days after transplantation. Representative confocal images from (**b**) corpus callosum white matter and (**c**) hippocampus. (**d**) U94A RFP+ hOPCs migrated significantly shorter distances than GFP+ controls. Cell dispersion was measured using stereological measurements of the distance of each cell to the center point of the transplanted cell population. Measurements averaged from at least three different sections from each of four different animals. (**e**) Analysis of NSG brains at 21 days post- transplant show that a majority of GFP+ and U94A RFP+ hOPCs differentiated into GSTπ positive oligodendrocytes (arrowheads). (**f**) No GFP+ or U94A RFP+ hOPCs were observed to differentiate into GFAP+ astrocytes (asterisks). Cell dispersion data displayed as mean ± SEM; *p < 0.05 versus GFP+ control. Effect of U94A expression on hOPC cell dispersion was analyzed by unpaired student’s *t*-test.

As impairment of hOPC migration leads to poor remyelination^4^, we wanted to confirm that the *in vitro* data are relevant in the complex *in vivo* environment of demyelinated tissue. To create a consistent injury with extensive myelin loss, we treated immunocompromised NSG mice with cuprizone for 4 weeks, leading to robust demyelination^15^. We then stereotactically transplanted U94A RFP+ and GFP+ control hOPCs contralateral into the corpus callosum and hippocampus (Fig. 3a). Twenty-one days after hOPC transplantation, brains were harvested and analyzed.

Consistent with our *in vitro* studies, we saw significant impairment in the migration of the U94A RFP+ cell pool compared to the control GFP+ cells in both the hippocampus (Fig. 3b) and the corpus callosum (Fig. 3c). Most U94A RFP+ cells remained as a bolus, while GFP+ cells dispersed widely throughout both white and gray matter (Fig. 3b,c). Quantification of cell spread of the U94A RFP+ and GFP+ hOPCs (Fig. 3d) showed a significant difference in their migration after transplantation of equal numbers of cells into each brain (p = 0.0178, unpaired t-test).

We also examined whether U94A expression altered the differentiation potential of transplanted hOPCs *in vivo*. We found that both U94A RFP+ cells and GFP+ controls generated GST-pi+ oligodendrocyte progeny (Fig. 3e) while neither of the cell populations were found to contain GFAP+ astrocytes (Fig. 3f). This finding is to be expected, as previous murine transplant studies have demonstrated that even at 8 weeks post injection, fewer than 5% of human cells express GFAP^14^. While > 90% of the GFP+ cells expressed GST-pi, we could not clearly quantify the number of U94A RFP+ cells that also stained for GST-pi, due to the impaired migration and clumping of the cells at the injection site.

## Discussion

In conclusion, (i) infection of hOPCs with HHV-6A is associated with the expression of the latency viral protein U94A and (ii) expression of the latency-associated viral protein U94A is sufficient to decrease the migration of hOPCs *in vitro* as well as *in vivo*. Our data raise the possibility that CNS viral latency is not a benign state, but can contribute to defective myelin repair through impaired recruitment of myelinating OPCs after injury. These data suggest, for the first time, a potential contribution of latent HHV-6A infection in the progression of demyelinating diseases.

This view offers a significant paradigm shift from previous approaches that focused on the effects of *primary* HHV-6A infection on CNS cell types and which have attempted to link the presence of HHV-6A with disease initiation^16^. Our hypothesis that HHV-6A latency may cause inefficient OPC-mediated repair in patients with chronic demyelinating disease is consistent with both evidence of HHV-6 viral infection but an absence of infectious virions in demyelinated lesions^12, 17^ and a failure of OPCs to populate these lesion sites^4, 5^. We therefore propose that the expression of viral transcripts and proteins during HHV-6A latency may contribute to progression of chronic demyelinating diseases. Lastly, our findings have important implications for the use of hOPCs for therapeutic purposes and the need to screen for and exclude the presence of HHV-6 in cell transplants.

## Methods

### Human Oligodendrocyte Progenitor (hOPC) Isolation

Human OPCs were isolated and maintained as previously described^18^. Briefly, cortical tissue from 19–21 week old fetal brain was dissected and the minced tissue was digested at 37 °C with 59 U/ml papain (Worthington) in Hanks balanced salt solution (HBSS, Invitrogen) supplemented with 10 mM Hepes (EMD), pH 8.0 and 125 U/ml Dnase I (Sigma), and triturated in 0.3%(w/v) BSA/HBSS (Sigma), 250 U/ml Dnase I. CD140a^+^ human oligodendrocyte progenitor cells (hOPC) were isolated by immunopurification using anti- CD140a^+^ bound magnetic beads (Miltenyi). Cells were plated on tissue culture plastic coated with poly-L-lysine (1 μg/cm^2^ for 20 min; Sigma #P1274) at a density of 5000 cells/cm^2^ in DMEM:F12 (Gibco #11330–057) supplemented with 10 μg/mL insulin (Sigma #I5500), 100 μg/mL holotransferrin (Sigma #T2252), Sato media (final concentration: 0.03% BSA Fraction V [Sigma #A7979-50ML], 10 μM putrescine [Sigma #P7505], 200 nM progesterone [Sigma #P0130], 235 nM sodium selenite [Sigma #S1382]), 50 μg/mL gentamycin (Gibco #15750–060), 10 ng/mL PDGF-AA (R&D #221-AA), and 10 ng/mL basic FGF (Miltenyi #130–093) and maintained at 37 °C (5% O_2_/7% CO_2_). When cells reached a density of 13000 cells/cm^2^ they were passaged with 0.05% trypsin-EDTA (Gibco #2300), neutralized with 80 Kunitz/mL soybean trypsin inhibitor (Sigma #T9003), and replated in DMEM:F12 complete media supplemented with 10 ng/mL PDGF-AA and basic FGF. Cells are maintained in this media for the remainder of their time in culture, unless exposed to differentiation conditions.

### Human herpesvirus 6 A (HHV-6A) Propagation and hOPC Infection

HHV-6A virus stocks from the U1102 strain were produced by serial propagation in permissive human lymphoblastoid cell lines and cell-free virus was prepared as we described previously in Dietrich *et al*.^9^. For infection of hOPCs, 200 μl of the cell-free virus preparation was added to hOPCs suspended in 800 ul of DMEM:F12 complete media supplemented with 10 ng/mL PDGF-AA and basic FGF. Following a four hour incubation at 37 °C (5% O_2_/7% CO_2_), the cells were replated in DMEM:F12 complete media supplemented with 10 ng/mL PDGF-AA and basic FGF. At four and ten days post infection, RNA was isolated from the hOPCs using a Nucleospin RNA kit (Machery-Nagel #740955) as per manufacturer’s protocol. 70 ng of RNA from each sample were converted to cDNA using an iScript cDNA Synthesis kit (Bio-Rad #1708890) with the addition of RNasin Plus RNase inhibitor (Promega N261B). For PCR analysis of U94A transcript expression, 5 μL of each cDNA sample was transferred to 50 μL nested PCR reactions as previously described^19, 20^.

### U94A Viral Propagation and hOPC Infection

HHV-6A cDNA was generated as above, and the U94A transcript was amplified and ligated into a RFP+ puromycin lentivector (System Biosciences #CD516B-1). Empty GFP lentivector (System Biosciences #CD513B-1) was used as a control. We used different fluorescent proteins to be able to clearly distinguish U94A+ cells from control cells. For viral production, 293 T cells were propagated in DMEM (Gibco #11965-02) with 10% fetal calf serum. Following co-transfection with Pax2 and VSV-G expressing plasmids, 293 T cells were maintained in 1% fetal calf serum for 72 h before harvesting viral supernatant. Infections of hOPCs were performed by adding lentiviral supernatant to cell cultures at a dilution of 1:4 in DMEM:F12 complete media supplemented with 10 ng/mL PDGF-AA and basic FGF. Cells were incubated at 37 °C (5% O_2_/7% CO_2_) for 4 h before media was changed.

### Analysis of hOPC Proliferation

The replication rate of the cells was determined by plating hOPCs on poly-L-lysine coated 96-well plates at a density of (300) cells/cm^2^ in DMEM:F12 complete media with 10 ng/mL PDGF-AA and basic FGF. The total number of hOPCs per well was determined using Brightfield analysis with a Celigo cytometer (Nexcelom) every other day across 21 days, and cell counts were normalized to the number of cells at the beginning of the experiment (Day 1).

### Analysis of hOPC Replication

The replication rate of the cells was determined by plating hOPCs on poly-L-lysine coated glass coverslips at a density of 1500 cells/cm^2^ in DMEM:F12 complete media with 10 ng/mL PDGF-AA and basic FGF. Three days after plating, the hOPCs were treated with bromodeoxyuridine (BrdU, Sigma #B5002) for 4–48 h then fixed with 2% paraformaldehyde. Coverslips were treated with sodium citrate buffer (10 mM sodium citrate, 0.05% Tween 20, pH 6.0) for 15 min using a steamer. Cells were permeabilized in 0.5% Triton X-100 and probed overnight at 4 °C with anti-BrdU mAb IgG_1_ (1:100, Sigma #B2531). Antibody binding was detected with appropriate Alexa Fluor-coupled antibodies at a concentration of 1 μg/ml (Thermo Fisher Scientific), applied for 1 h. All antibodies were diluted in HBSS (Invitrogen) plus 5% fetal calf serum with 0.5% sodium azide and 0.1% Triton X-100. Stained cells on coverslips were rinsed three times in 1 × PBS, counter-stained with 4′6-diamidino-2-phenylindole (DAPI; Thermo Fisher Scientific #D1306) and mounted on glass slides with Fluoromount-G (SouthernBiotech #0100-01).

### *In Vitro* hOPC Migration

Cell migration with agarose drops was performed as previously reported (Milner *et al*., 1997, Glia, p85-90) Briefly, U94A RFP or control GFP lentiviral infected hOPCs were mixed to equal parts, resuspended in 0.3% low-melt agarose (at 37 °C; Sigma #A0701), and diluted in DMEM:F12 complete media supplemented with 10 ng/mL PDGF-AA and basic FGF. at a density of /μL. 1.5 μL (containing 6 x 10^4^ cells) of the cell-agarose mixture was plated in the center of a poly-L-lysine-coated 24-well plate. The agarose was allowed to gel at 4 °C for 10 min before DMEM:F12 complete media supplemented with 10 ng/mL PDGF-AA and FGF. The number of U94A RFP+ and control GFP+ cells that had migrated from the edge of the agarose drop was quantified.

### *In Vitro* hOPC Differentiation

Experiments involved first expanding hOPCs in 10 ng/ml PDGF-AA and basic FGF growth factor (bFGF). At passage, cells were plated for experiments on poly-L-lysine coated glass coverslips at a density of 1500 cells/cm^2^ in DMEM:F12 complete media with 10 ng/mL PDGF-AA and basic FGF and allowed 24 h for recovery before treatment. Differentiation conditions consisted of 1 ng/ml PDGF-AA plus 40 nm thyroid hormone (TH) (30 ng/ml thyroxine and 36 ng/ml triiodothrionine) for 10 days to induce oligodendrocytes differentiation or 10 ng/ml bone morphogenetic protein 4 (BMP-4, R&D Systems #314-BP) for 7 days to induce astrocytes differentiation.

### Immunocytochemistry

Cells were fixed with 2% paraformaldehyde and then permeabilized in 0.5% Triton X-100 before probing overnight at 4 °C with anti-GFAP (1:1000, Agilent Technologies #Z033429-2) or anti-GalC hybridoma supernatant (1:4). Antibody binding was detected with appropriate Alexa Fluor-coupled antibodies at a concentration of 1 μg/ml (Thermo Fisher Scientific), applied for 1 h. All antibodies were diluted in HBSS (Invitrogen) plus 5% fetal calf serum with 0.5% sodium azide and 0.1% Triton X-100. Stained cells on coverslips were rinsed three times in 1 × PBS, counter-stained with 4′6-diamidino-2-phenylindole (DAPI; Thermo Fisher Scientific #D1306) and mounted on glass slides with Fluoromount-G (SouthernBiotech #0100-01). Images were acquired by LAS AF software using a Leica TCS SP5 laser confocal microscope (Leica Microsystems, Mannheim, Germany).

### Cuprizone Model and hOPC Transplants

All animal procedures were performed under guidelines of the National Institutes of Health and approved by the Institutional Animal Care and Utilization Committee (IACUC) of the University of Rochester Medical Center, Rochester, NY. Six month old male NSG mice were obtained from in-house breeding and fed with a diet of chow mixed with 0.2% cuprizone over the course of four weeks. Animal weights were recorded three times per week and care for those that lost > 15% body weight was discussed with a veterinarian. Animals showing inability to ambulate, inability to maintain food or water intake, and clinical signs of pain including ruffled fur, hunched posture, vocalization and guarding behavior were acutely euthanized. Handling of the animals when they were weighed was kept to a minimum and all animals were group housed to avoid isolation stress. For the transplantation procedure, mice were anesthetized with ketamine (100 mg/kg, i.p.) and xylazine (10 mg/kg, i.p.), and secured in a stereotactic frame (David Kopf Instruments, Tujunga, CA, USA). Six burr holes were drilled at 3 coordinates in each hemisphere (Bregma: mediolateral 1.5mm, anterior–posterior 0, 1.0, 2.0 mm, dorsoventral 0.9 mm). U94A RFP or control GFP-expressing hOPCs were resuspended in PBS at a concentration of 25,000 cells/μl. Two μl of U94A RFP or GFP cells were injected in contralateral hemispheres with a ten-μl syringe fitted with a glass capillary injection needle (80 µm diameter, beveled tip; pulled using a P1000 micropipette puller, Sutter Instruments).

### Immunohistochemistry

21 days following stereotactic cell injection, mice were transcardially perfused with 4% paraformaldehyde/PBS. Brains were isolated and post-fixed for 24 h in 4% paraformaldehyde and normalized for 48 h in 20% sucrose. Brains were frozen with dry ice and sectioned at 25-μm thickness using a cryotome. The sections underwent three washes in PBST (phosphate buffered saline + 0.03% Triton X-100) and were blocked in 5% fetal bovine serum serum in PBST (1 h). Sections were then immunostained overnight at 4 °C with GFAP (1:300; Sigma #G3893), and GSTpi (1:500; BD Biosciences #610718). The next day, the tissue was washed three times in PBST and incubated for 1 h in appropriate Alexa-Fluor-conjugated secondary at a concentration of 1 μg/ml (Thermo Fisher Scientific) and counter-stained with 1 μg/ml 4′6-diamidino-2-phenylindole (DAPI; Thermo Fisher Scientific #D1306). Stained sections were rinsed three times in 1 × PBS and mounted on glass slides with Fluoromount-G (SouthernBiotech #0100-01). Multi-channel fluorescence mosaic images were acquired by LAS AF software using a Leica TCS SP5 laser confocal microscope (Leica Microsystems, Mannheim, Germany). Images were acquired with a 40 × oil immersion lens (Leica) and *z*-projections of 6 images taken at 7-μm step intervals.

### Cell Spread Measurements

Migration data represent analyses of the corpus callosa and hippocampi of four animals. A minimum of three sections per animal were analyzed, and only sections containing both GFP+ and U94A RFP+ cells were used for analysis. To measure cell spread, coordinate positions of each cell were determined and the distance of each cell from the mean coordinate position of the cell population was measured using LAS AF software (Leica Microsystems, Mannheim, Germany).

### Statistics

Bar graphs are plotted as mean ± SEM and represent, at minimum, three independent biological replicates performed in triplicate. Two-group comparisons were analyzed using a Student’s *t* test, and multiple-timepoint comparisons were analyzed by two-way ANOVA. Prism (v5.0; GraphPad) was used for data analysis and presentation.

### Ethics Statement

All animal procedures were performed under guidelines of the National Institutes of Health and approved by the Institutional Animal Care and Utilization Committee (IACUC Protocol Approval number #100895/2001-240) of the University of Rochester Medical Center, Rochester, NY Human tissues were obtained from de-identified cadaver specimen using the Safe-Harbor Method that is not subject to informed consent. All procedures were reviewed and approved by the University of Rochester Research Subjects Review Board (RSRB), RSRB No. RSRB00024759.

## Electronic supplementary material

Supplementary information
