# Supplementary material for: Expression of the Human Herpesvirus 6A Latency-Associated Transcript U94A Disrupts Human Oligodendrocyte Progenitor Migration
Source: Sci Rep. 2017 Jun 21;7:3978. doi: 10.1038/s41598-017-04432-y (PMC5479784; doi:10.1038/s41598-017-04432-y)

Expression of the Human Herpesvirus 6A Latency-Associated Transcript U94 Disrupts Human Oligodendrocyte Progenitor Migration

Campbell A[#], Hogestyn J[#], Folts C, Lopez B, Proschel C, Mock D and M. Mayer-Proschel*

Supplementary Information – Figure 2a U94A nested PCR

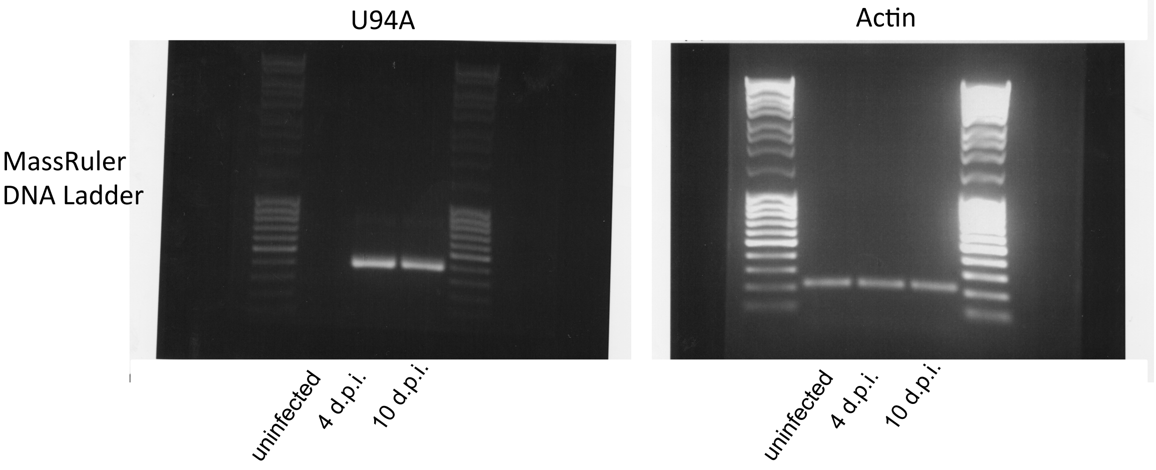

Supplement: Supplementary file 1 — Supplementary information [file 41598_2017_4432_MOESM1_ESM.pdf]
